# Reducing the Brace Correction Stress on the Secondary Lumbar Curve Results in Excellent Muscle, Bone, and Disc Mechanical Performance: A Musculoskeletal Finite Element Simulation of AIS Patient With Rigo A3

**DOI:** 10.1111/os.14296

**Published:** 2024-11-12

**Authors:** Xiaohui Zhang, Di Wang, Danyu Lv, Jinmiao Lv, Huiyi Tang, Jinlin Qian, Bagen Liao

**Affiliations:** ^1^ Department of Sports Medicine Guangzhou Sport University Guangzhou China; ^2^ Guangdong Provincial Rehabilitation Engineering Center for Adolescent Idiopathic Scoliosis, Guangzhou Sport University Guangzhou China; ^3^ Guangdong Provincial Key Laboratory of Physical Activity and Health Promotion, Guangzhou Sport University Guangzhou China

**Keywords:** downward correction pressure, finite element simulation, improved performance, lumbar spine curve, tensegrity

## Abstract

**Objectives:**

The biomechanical mechanism of brace intervention on bone, muscle, and disc should be comprehensively considered for AIS patients. We aimed to developmentally construct a musculoskeletal finite element model of adolescent idiopathic scoliosis to simulate the coupling of corrective forces and analyze the mechanical properties of bone, muscle, and disc. Investigateing, more effective clinical interventions to break the vicious cycle of patients during growth.

**Methods:**

A finite element model, including muscle, bone, and disc, was established using computed tomography data of a patient with RigoA3 adolescent idiopathic scoliosis. The three‐point force coupling, antigravity, and bending effects of the Chêneau brace were simulated, and the correction force of the secondary lumbar bend was gradually reduced while observing the mechanical characteristics of bone, muscle, and disc. The correction force in line with symmetrical spine growth was comprehensively evaluated.

**Results:**

The correction rate of the main thoracic (MT) curve, the intervertebral space height on the concave side of the vertebrae at the apex, and the stress ratio of the intervertebral discs were optimal when the maximum corrective pressure threshold was reached. However, the proximal thoracic (PT) curve was aggravated and the axial forces on the concave side were unbalanced. At this time, the biomechanical performance of the model is also not optimal. The correction rate of the Cobb Angle of the MT curve decreased with the decrease of the correction pressure in the lumbar region. When reduced to 25% of the maximum threshold, the convex side of disc stress, intervertebral space, and muscle axial force is more in line with the biomechanical mechanism of correction and can avoid sacrificing the PT curve.

**Conclusions:**

Downward adjustment of the corrective force to the secondary lumbar curve, using the Chêneau brace, results in better primary thoracic curvature mechanics in the musculoskeletal finite element model, suggesting that breaking the vicious cycle of scoliosis progression to guide benign spinal growth is beneficial.

## Graphical abstract

A Rigo A‐classified finite element model of adolescent idiopathic scoliosis was constructed, which included muscles, bones, and intervertebral discs conforming to the tensegrity of the spine. Reducing the corrective force of the compensatory lumbar curve of Rigo‐Chêneau brace results in preferable radiological parameters, muscle axial force, and intervertebral disc height and stress balance, which would be beneficial to break the vicious cycle of deformity during growth.

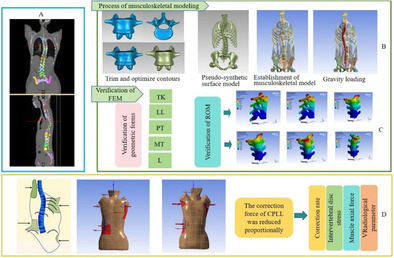

## 1 Introduction

Adolescent idiopathic scoliosis (AIS) is a three‐dimensional, structural deformity of the spine, defined as a radiological lateral Cobb angle ≥ 10°. AIS affects 1%–3% of children (aged 10–16 years) at or around puberty, and a proportion of children with AIS require corrective intervention to control the progression of the scoliosis [1]. Patients with AIS usually present with uneven shoulders, waistline asymmetry, or rib prominence [2]. Although most AIS curves do not require surgical intervention, a Cobb angle of ≥ 30 poses the most health risks, especially in skeletally immature patients [3]. Health risks include back pain, mental health issues related to appearance [4], and cardiac risks [5]. Although the etiopathological mechanisms of curve occurrence and progression of AIS remain unclear, biomechanical mechanisms are involved in the development of asymmetric spinal malformations.

Stokes' vicious cycle states that spinal deformity produces asymmetrical spinal loads that induce asymmetrical growth in patients with AIS. The spine deformity results from a combination of disc and vertebral wedging. However, the relative contributions of these two structures are not well defined [5]. A longitudinal study of patients with progressive AIS demonstrated that spinal deformities begin with intervertebral disc (IVD) changes, and wedging of the IVDs precedes wedging of the vertebra during the growth spurt [6]. Disc torsional deformation causes greater anterior overgrowth and coronal wedging in the discs than in the vertebral bodies [7]. Furthermore, mechanobiological studies demonstrated significantly greater disc height asymmetry in patients with AIS, suggesting that excessive disc pressure on the concave side leads to annular fibrosis and longitudinal ligamentous tension, which may lead to the inability to remodel and grow. Differences in IVD pressures on the concave and convex sides may drive the development and progression of AIS [8].

Bracing is the most effective treatment for the patients with mild to moderate AIS [9]. AIS patients with immature skeletons are at the greatest risk of curve progression, particularly those with a Cobb angle ≥ 30°. The goal of bracing is to prevent the progression of the curve to a severity that requires surgery [3]. The Rigo‐Chêneau brace, which is frequently recommended to control curve progression, exhibits excellent clinical results [10, 11]. In clinical studies, the brace resulted in a 72% success rate, indicating that many patients with AIS are still at risk of progression, even with the brace [12].

Recently, the biomechanical mechanism of bracing has been investigated to improve the predictability of brace correction results. Finite element models (FEMs) have been applied to the biomechanical studies of scoliosis treatment [13].

Furthermore, computer‐aided design (CAD) using FEMs has been proposed as a time‐ and cost‐efficient approach for managing scoliosis. These models have resulted in promising brace corrections and improved clinical satisfaction outcomes [14, 15]. However, little research has focused on musculoskeletal and finite element studies of patients with AIS. Previous studies revealed that the center of reaction the deviates from the IVD, resulting in uneven stress distribution on the IVD disc and increased reaction torque in FEMs [16]. Stress changes in the disc affect the growth of the scoliosis curve, which affects the final correction result.

FEMs have been used to simulate the correction effects of braces and optimize the design and management of braces [17, 18]. However, brace corrections involve the coupling of multiple three‐dimensional three‐point forces rather than a simple single plane. Moreover, few finite element simulation studies have focused on how brace corrections change the stress characteristics of the concave and convex sides of the IVD. The Chêneau brace controls the curve of scoliosis, but some patients are still at risk of correction failure, especially in the thoracic curve [19]. Our previous studies showed that when there are two curves, lowering the corrective stress of the secondary curve results in a superior correction rate for the primary curve [20]. This mechanism may also influence the height and stress of the discs involved in differential growth. Therefore, the purposes of this study included: (i) To developmentally construct a musculoskeletal FEM of spinal tensegrity for AIS. (ii) The biomechanical responses of bone, muscle, and IVD to different bracing force combinations were comprehensively measured. (iii) Investigating a combination of brace correction forces aimed at improving clinical predictability that optimizes biomechanical performance of muscles and IVDs to help break the vicious cycle of malformations.

## 2 Materials and Methods

This study was approved by the Human Subject Committee of Guangzhou Sport University (2021LC LL‐16), and informed consent was obtained from the patients or parents of minors.

Musculoskeletal models of trunk muscles are based on the coordinates of the muscle fascicle attachment points [16]. Muscle reaction moments, which are calculated using musculoskeletal models, and gravity loads and muscle forces after Chêneau brace correction force simulation were prescribed using patient‐specific FEMs to estimate the stress distribution within the T1 to L3 IVDs. The Cobb angles and Cobb angle correction ratios were also analyzed.

### 2.1 Data Acquisition

A 13‐year‐old female patient with Rigo A3 scoliosis, a height of 152 cm, and a weight of 42 kg was included in the study. A Siemens dual‐source computed tomography (CT) scanner (Siemens Somatom Definition 2012B; Siemens Healthineers, Erlangen, Germany) was used for transverse scanning of the entire spine and pelvis, covering T1 through S1, with a layer thickness of 0.7 mm. All CT images were saved in DICOM format.

### 2.2 Building the Three‐Dimensional Musculoskeletal Model

**FIGURE 1:**
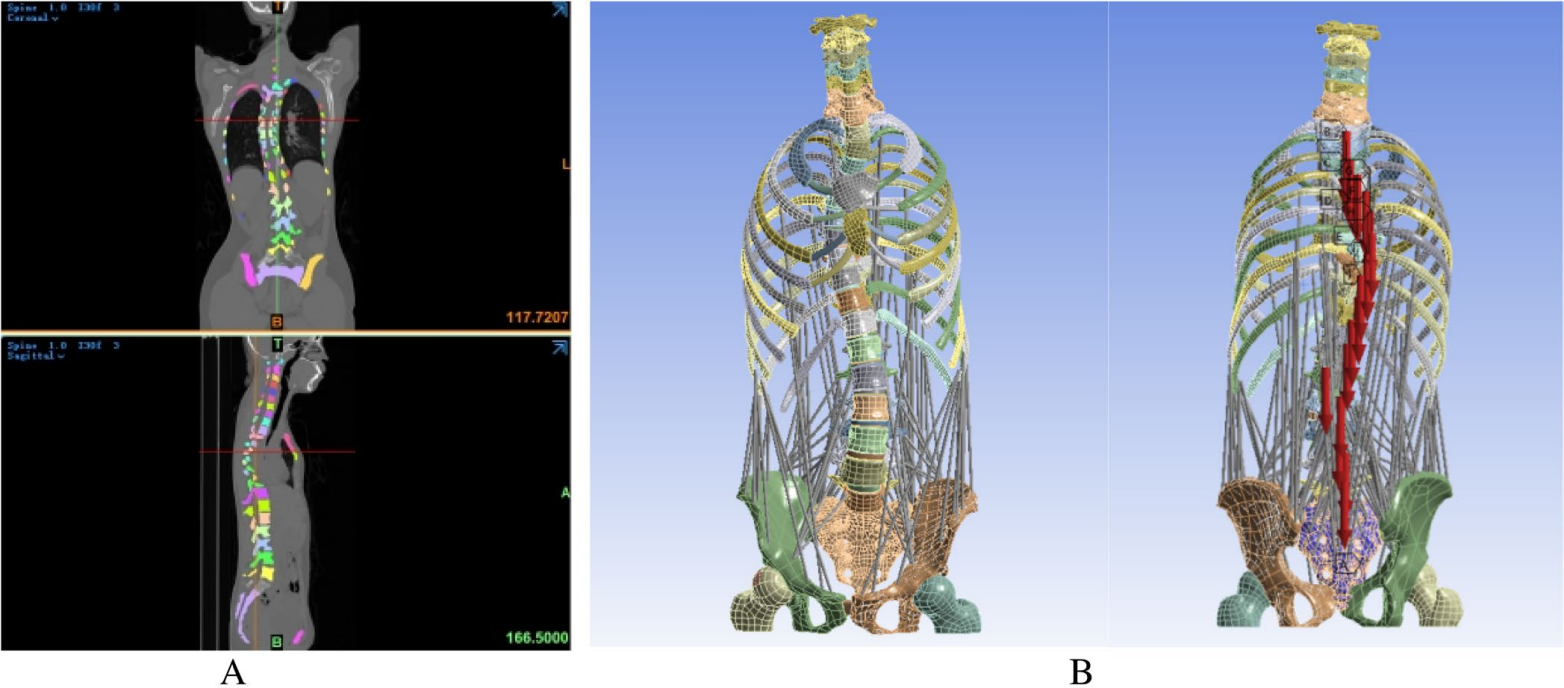
The musculoskeletal finite element model was constructed for Rigo A classification. (A) Radiographic data of the spine and pelvis were obtained by CT. (B) The skeleton of the model was optimized and the whole spine‐pelvis musculoskeletal assembly model was established.

The DICOM format CT images were imported into Mimics 21.0 software (Materialize, Leuven, Belgium) [18]. The model coordinates were defined as follows: coronal axis, *X*; sagittal axis, *Y*; and horizontal axis, *Z*. The cervical, thoracic, lumbar, sacral, and pelvic vertebral regions were identified based on the gray values of different tissues. The identified bones were imported into Geomagic Studio 2017 (Geomagic Inc. Research Triangle Park, NC, USA) in STL format to process the uneven surface of the medical image accumulation (Figure 1A). Each surface component was imported into SolidWorks 2020 (SolidWorks Corporation, MA, USA) to create a solid individual model fitting for the spine‐pelvis assembly [18]. The models included the articular cartilage, cortical bone, cancellous bone, fibrous annulus, nucleus pulposus and endplate, and muscle. The attachment positions of muscle bundles and bones in the FEM were added based on the classic skeletal muscle model. The muscle was idealized based on previous reports, and the physiologic cross‐sectional area of the skeletal muscle was determined to establish the trunk muscle model [21, 22, 23, 24, 25, 26, 27, 28, 29]. The curve of the whole extensor muscle [30] and the curve of the internal and external oblique muscles of the abdomen were simulated using the elliptical torso model [27]. Finally, the models were combined and saved in the SLDPRT part format.

Ansys Workbench 19.0 was used to perform the finite element analysis (ANSYS Inc. Cannon Sburg, USA). To ensure the accuracy and validity of the model and optimize the demand for computing resources, the solid model of each vertebra was divided into a high‐quality mesh using the adaptive dynamic mesh of the modeler. Bone, cartilage, and other tissues, including the anterior longitudinal, posterior longitudinal, Ligamenta flavum, supraspinous, and interspinous ligaments, were specified as the tetrahedral solid units of a network using Ansys Workbench 19.0. Spring elements were used to simulate ligaments between the vertebrae (Figure 1B). All components were defined as elastomers, and the bone structure and discs were modeled as isotropic elastic materials. The material properties of bone, IVD, and ligament were determined based on experimental literature [16, 17, 21, 31, 32, 33, 34, 35, 36]. The material parameters of bone and soft tissue are summarized in Table 1 [15]. Different materials can be distinguished according to their differences in elastic modulus and Poisson's ratio.

**TABLE 1:** Material properties of each part in the finite element model (FEM).

Competent name	Elastic modulus (MPa)	Poisson's ratio (*v*)
Cortical bone	12,000	0.30
Cancellous bone	100	0.20
Posterior structure of vertebral body	3500	0.25
Nucleus pulposus of intervertebral disc	1	0.49
Annulus fibrosus of intervertebral disc	4.2	0.45
Rib	5000	0.10
Sternum	9592	0.20
Pelvis	5000	0.20
Sacrum	5000	0.20
Costal cartilage	300	0.20
Ligament	31.5	0.45
Skin	31.5	0.42

**FIGURE 2:**
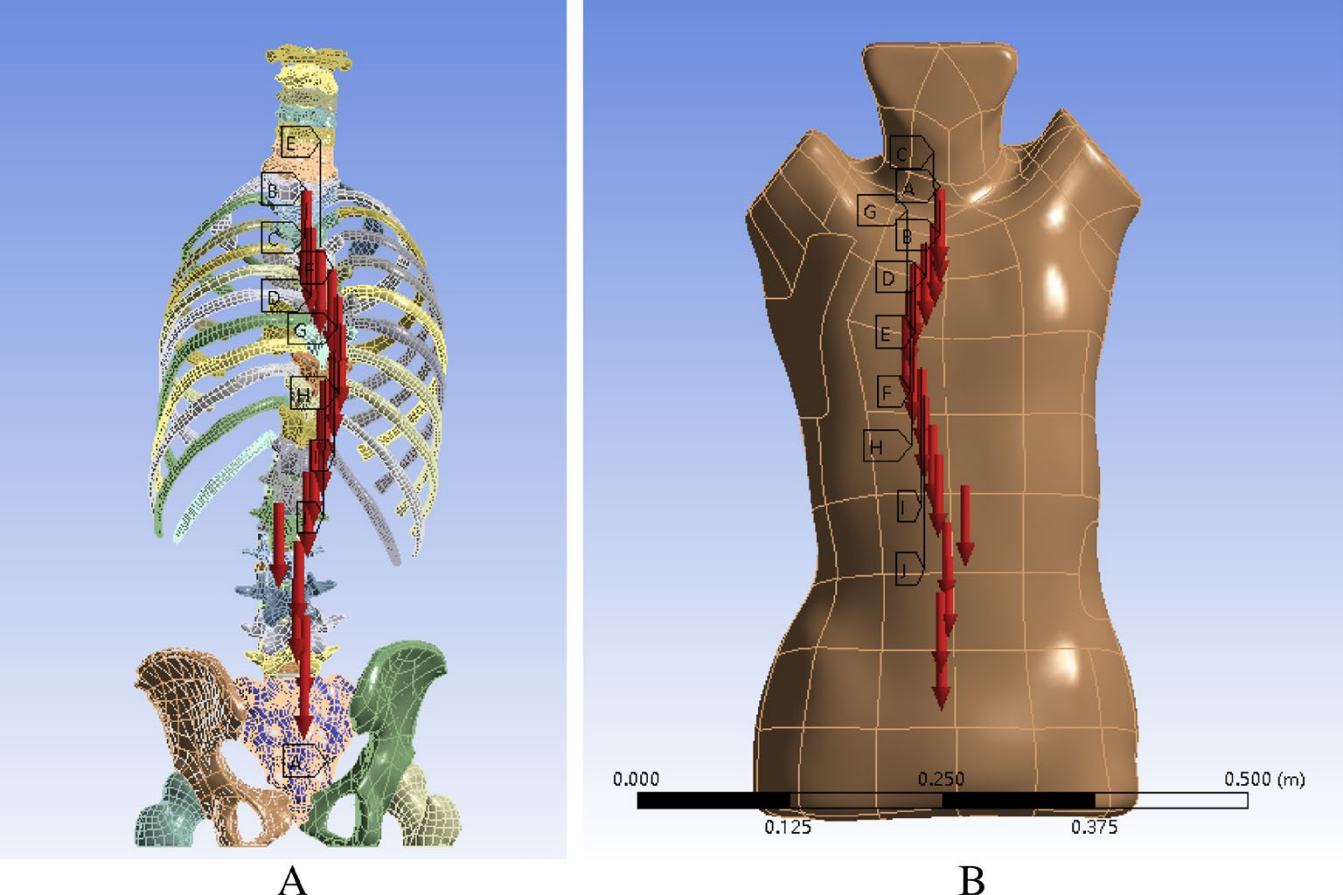
Red arrows represent the direction of gravity and muscle force application, (A) represents the mixing force, C–K (B) represents magnitude of the applied force.schematic diagram of loads at different vertebral levels under FEM simulated gravity and muscle factors (spine–plevis FEM) vertical and downward gravity load is applied to the surface of the vertebrae.

To observe the important effects of muscles and ligaments on the stability and mobility of the spine, a muscle ligament model was constructed. Muscle forces and reaction moments were estimated in the upright‐standing posture under load gravity simulation, which was constrained to equilibrium and stability conditions, and the scoliotic curvature and stress distribution were calculated (Figure 1B). According to Hueter–Volkmann's law, increased pressure on the concave side of the epiphysis and increased tension on the convex side change the growth rate of the vertebral body, manifested by concave inhibition and convex acceleration [37]. Loading on the spine includes gravitational forces acting on the vertebrae and muscle forces that maintain spinal stability [38]. Based on previous studies [39, 40], we assumed that the load on the vertebral body is closely related to the body's self‐gravity. The musculoskeletal FEM of the AIS‐simulated forces in the upright‐standing position of the patient is shown in Figure 2. The actual weight of the patient with AIS was 42 kg. The simulated forces act along the *Z*‐axis at the center of the vertebral body, constraining all degrees of freedom of the sacrum. The simulated forces allow T1 forward flexion, lateral flexion, and vertical translation to maintain balance. The equation used to calculate the loads on the patient at each vertebral level [20] is shown below.

$$\text{Ptot}i=\left(15+2.1\times \text{Mass}i\right)$$

$$\Delta \left(\text{Ptot}i\right)=\text{Ptot}i-\text{Ptot}i–l$$

$$\Delta \left(\text{Force}i\right)=\Delta \left(\text{Ptot}i\right)\times g$$

where *g* = 9.81 m/s; Mass*i*, mass corresponding to the *i*th vertebral body level; Ptot*i*, cumulative mass of the part above the level of vertebral *i*.

### 2.3 Mesh Sensitivity Analysis

Sensitivity analysis was performed after each parametric FEM was generated. The reason for the analysis is to determine the correct size of the part, as well as the type of part used and its numerical formula to ensure excellent results at the lowest computational cost [41, 42]. According to the previous study on the FEM of scoliosis, Ansys Workbench 19.0 was used to divide tetrahedral elements. We selected T7 as the research object to divide the number of four groups of mesh and conduct axial load simulation analysis [43]. It can be seen from Table 1 that the number of meshs has a corresponding impact on the results.

When the number of meshs is < 20,215, the difference of results is obvious. With the increase of the number of meshs, the stress–strain of the vertebrae divided by the number of adjacent meshs also decreases. The difference between the number of grids D and the number of grids C is the smallest, The difference between stress and displacement is 4.37% and 0.05%, respectively. Therefore, it can be considered that Group C satisfies the verification.

**FIGURE 3:**
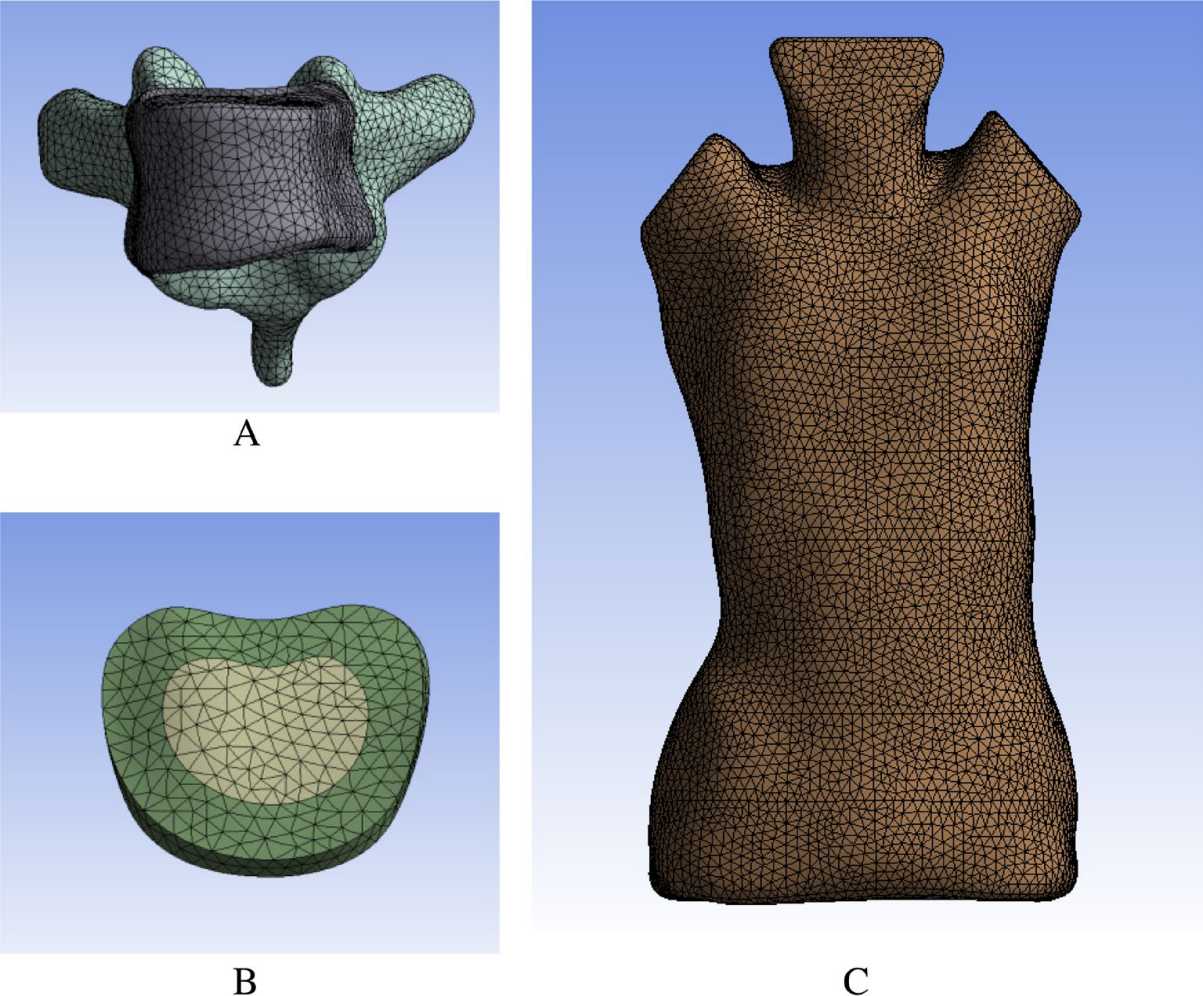
Schematic diagram of the meshs, where (A) is the vertebra, (B) is the disc, and (C) is the torso and pelvis.

Therefore, it can be considered that in Group C, the meshes number of the vertebral body is 29,308, which meets the verification. The meshs quality check criteria are as follows: aspect ratio ≤ 5°, maximum Angle ≥ 30°, minimum angle ≤ 120°, Jacob ratio > 0.7, warpage ratio ≤ 0.6.At this time, the meshs of vertebrae and the whole torso is shown in Figure 3A. The overall torso meshes model is shown in Figure 3C.

### 2.4 Loading and Boundary Conditions

The intermodel contacts were defined based on the structure and motion characteristics of the spinal–pelvic region. The types of contact between models were defined in the connections. The contact type of the joint surfaces was defined as no separation, which does not allow the surfaces of the contact area to separate but allows small frictionless sliding along the contact surface. High pressure on the body causes discomfort and pressure ulcers. A reasonable stress range and area were selected to ensure acceptable local pressure generation by the body brace. Modifications due to human gravity and muscle forces were comprehensively considered, and correction forces within the range of the human pain threshold were guaranteed. Therefore, the corresponding ceiling thresholds for the area and correction force were set according to the model previously reported in this study (Table 2) [44, 45].

**TABLE 2:** Area and force direction of the brace pressure region simulated by the FEM model.

Pressure area location	Pressure area location (cm^2^)	The direction of force
Left arm	38.5	From ventrolateral to dorsomeally
Left waist	116.0	From the dorsolateral to the ventromedial direction
The right chest	143.9	From the dorsolateral to the ventromedial direction
Left underarm	47.1	Vertical up
On the right arm	23.5	Vertical up

### 2.5 FEM Validation

Number of grids influences the results of the numerical calculation. Improving the grid quality gradually reduces the performance prediction error [46]. When grid increases have little influence on the calculated results, the numerical results are meaningful [17]. The model was meshed to ensure the accuracy of the calculations. The grid type was a tetrahedral mesh, and the grid size of the joint surfaces was set to 0.8 mm; the other grids were set at 1.5 mm. Therefore, the model was divided into 1,353,236 elements and 2,381,761 nodes.

**FIGURE 4:**
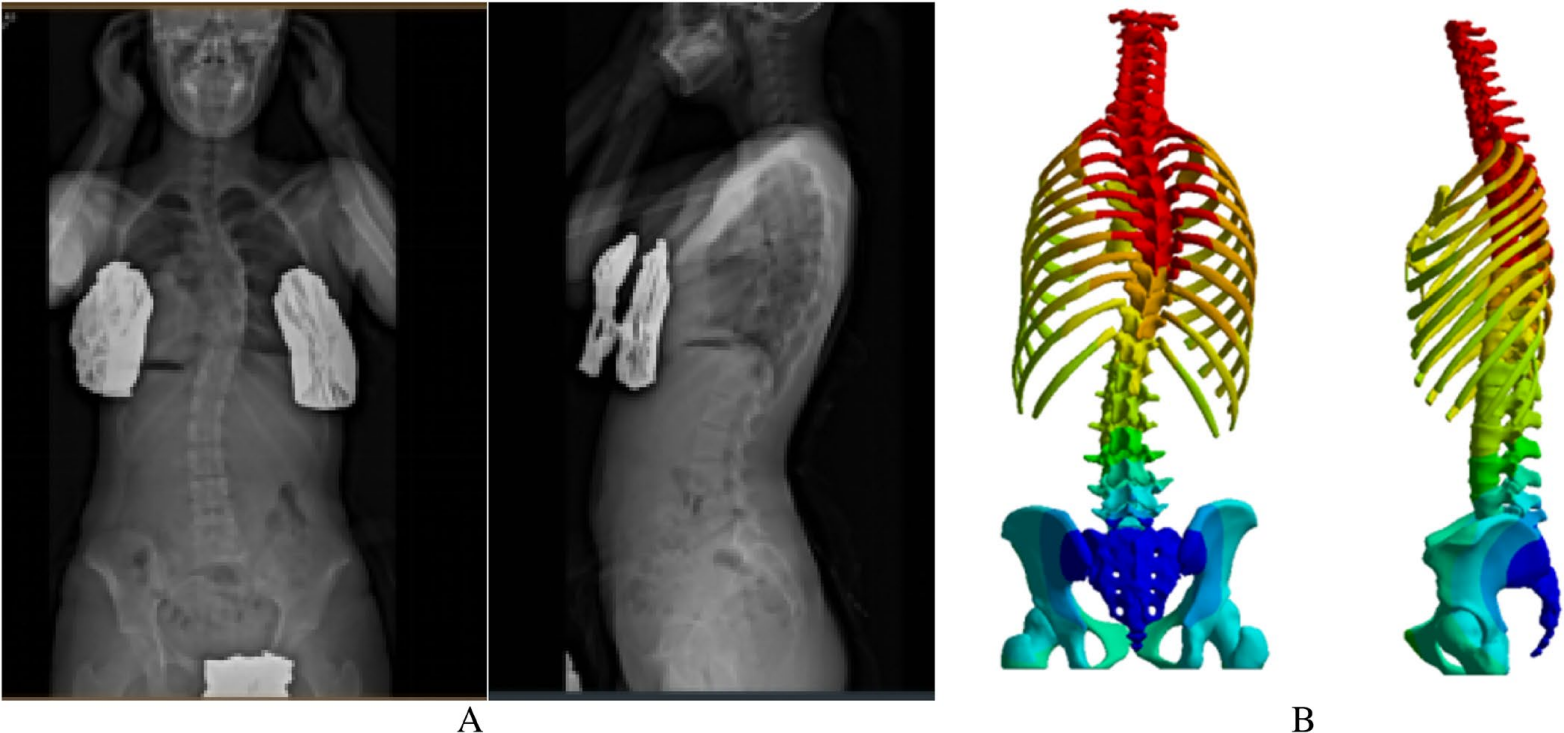
Comparison of the geometric shape of the patient's full spine anterior‐lateral radiographs with the finite element model.

The biomechanical properties of the FEM were validated to ensure the reliability of the mechanical response analysis during the brace correction simulation [47]. The geometry, range of motion (ROM) of the vertebral segments, and muscle axial force of the model were validated. To verify the geometric similarity of the model, the spinal imaging parameters of the model were measured after gravity loading (Figure 4). The model was adjusted to the coronal plane, and the Cobb angles of the main and near chest bends were measured using the x‐ray sheet Cobb measurement method. The model was adjusted to the sagittal plane to measure the thoracic kyphosis (TK) and lumbar lordosis (LL) angles. The model was adjusted to the axial position, the vertebra T2 and T7 were located, the respective axial vertebral rotations were measured, and the measured values were compared with the parameters from the patient's EOS imaging. The numerical difference between this model and the clinical imaging parameters was < 5°. Therefore, this model exhibited geometric similarity with the clinical data (Table 3).

**TABLE 3:** Radiological parameter measurements were compared between the finite element model and the x‐ray examination.

Parameters	MT	PT	L	TK (°)	LL (°)
Radiological measurements	38.5	19.8	34	10	40.5
FEM Model	39.6	22.5	33	11	42.4

Abbreviations: L, lumbar curve; MT, main thoracic curve; PT, proximal thoracic curve.

**FIGURE 5:**
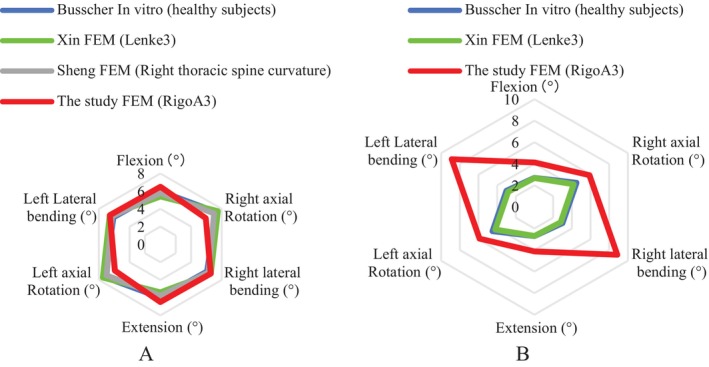
Comparison of vertebral segment ROM with previous studies. (A) Comparison of ROM from T1 to T4. (B) Comparison of ROM from T1 to T4.

The ROMs of the T1–T8 joints in this model were previously verified. All degrees of freedom were constrained on the lower surface of the T4 vertebra, and 4 Nm torques were applied to the upper surface of the T1 vertebra. Forward flexion and backward extension, left and right lateral flexion, and axial rotation were simulated to verify the T1–T4 segment ROM. Similarly, the ROM of T5–T8 in the model was verified [48, 49, 50]. The T1–T4 segment ROM simulation results were verified (Figure 5A); the stimulated ROM values in this model were consistent with anterior flexion, dorsal extension, left/right flexion, and left/right rotation ROM values from previous research models [48, 49, 50]. However, structural changes near the chest bend differed in this model; the left/right ROM values were slightly lower than the ROM values in the three preceding models but were within an acceptable range. Verification of the T5–T8 segment ROM (Figure 5B) revealed that the anterior flexion values and left/right rotation ROM simulated in the selected segments of this model were higher than those values obtained by Busscher et al. [48] and Xin et al. [50]. The location of the top of the main thoracic (MT) curvature, the different degrees and types of lateral curvatures, and the older age in the previous models may make the ROM values of this model slightly larger than those of previous models.

Herein, the musculoskeletal model incorporated an optimization algorithm based on balance and stability conditions to measure the axial force of muscles in the upright‐standing position and compared the overall concave/convex ratio of the axial force of muscles, including the concave–convex multifidus and longissimus muscles, with the musculoskeletal model of Kamal and Rouhi [51]. In our model, the ratios of the multifidus were 0.95 for the concave–convex muscle, 0.82 for the longissimus psoas, 0.89 for the longissimus pectoris, 0.75 for the quadratus psoas, and 0.86 for the psoas major. The overall ratio was 0.86, which was close to the concave/convex ratio of the paravertebral muscle group in the previous FEM (0.88). In summary, the new model is reliable and complies with the biomechanical simulations.

### 2.6 Boundary and Loading Conditions for Brace Correction FEM Simulation

**FIGURE 6:**
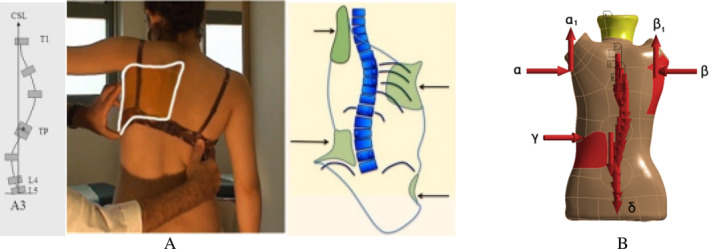
Correct force finite element model of Chêneau brace Simulate the correction mechanism according to the concept of the Chêneau brace. (A) Arrows indicate the direction of the applied correction force and the red areas represent the area of the corrected force applied. (B) The direction of applied stress force is as follows: α represents a thrust applied on left axillary lateral (α₁ represents upward axillary extension inferior), β represents a thrust applied on Right thorax lateral (β₁ represents‐upward axillary extension inferior), γ represents a thrust applied on Left lumbar posterior, and δ represents gravity and muscle force applied on trunk. The model's pelvis and sacral canals were restricted to *X*, *Y*, and *Z* axis of freedom, and T1 was allowed to do lateral bending, forward bending and upward extension.

The Chêneau brace provides space for tissue displacement, growth, and respiratory movement by applying corrective force on the convex side of the spine and releasing pressure on the concave side. According to the correction mechanism of the Chêneau brace, the model's pelvis and sacral canals were restricted to *X, Y*, and *Z* axis of freedom, and T1 was allowed to do lateral bending, forward bending, and upward extension. Previous studies [52, 53] have shown that the corrective thrust was applied to the right chest to correct the right thoracic curve, the corrective reaction force was applied to the left axilla and the left lumbar side, and an upward extension force was applied to the axillae (400 N on the left and 200 N on the right) to simulate the “bending effect” and the “antigravity effect” of the brace (Figure 6).

The FEM was used to simulate the corrective force coupling of the brace in three regions of the torso, including the left axilla, left lumbar, and right lateral thoracic region (Table 4). The correction pressure of the left lumbar region (CPLL) was scaled down gradually, and the effects of the downward adjustment of the CPLL were simulated based on the three‐point correction force principle of the brace. According to our previous clinical study [20], lowering the correction pressure for the compensatory lumbar curvature induces a better correction rate for the MT curve. Therefore, in the simulation, the CPLL was reduced by 25%, 50%, 75%, and 100%, and the biological characteristics of the correction according to the maximum threshold setting were observed in other areas. The internal pressure threshold for the trunk tolerance was 0.035 MPa [45] and the axillary brace tolerance was 0.085 MPa [54]. Using these pressure threshold values, four different correct pressures were set (Table 3), including correct finite model element (CFME)1, CFME2, CFME3, and CFME4. The effective pressure area was determined based on previous studies, patient conditions, and clinical experience [38, 55]. The area of the correction force varied in different body parts (Table 4).

**TABLE 4:** The area and direction of the correcting force of the Chêneau brace are simulated.

Combination groups	Left axillary	Left lumbar	Right thorax	Left axillary inferior	Right axillary inferior
CFEM1	0.035	0.009	0.035	0.085	0.085
CFEM2	0.035	0.018	0.035	0.085	0.085
CFEM3	0.035	0.026	0.035	0.085	0.085
CFEM4	0.035	0.035	0.035	0.085	0.085

### 2.7 Biomechanical Analysis of the Brace Correction Simulation

The relevant parameters for the mechanical response of the corrected model were measured. First, the Cobb angle, TK, LL, and apical vertebral translation (AVT) were measured radiologically. The AVT in the proximal thoracic (PT) and MT regions were recorded as AVT_PT_ and AVT_MT_, respectively. The correction rate for the Cobb angle was calculated using the following formula:

$$\text{Correction rate}\;\left(\%\right)=\frac{\text{prebrace Cobb Angle}-\text{inbrace Cobb Angle}}{\text{Prebrace Cobb Angle}}\times 100\%$$

**FIGURE 7:**
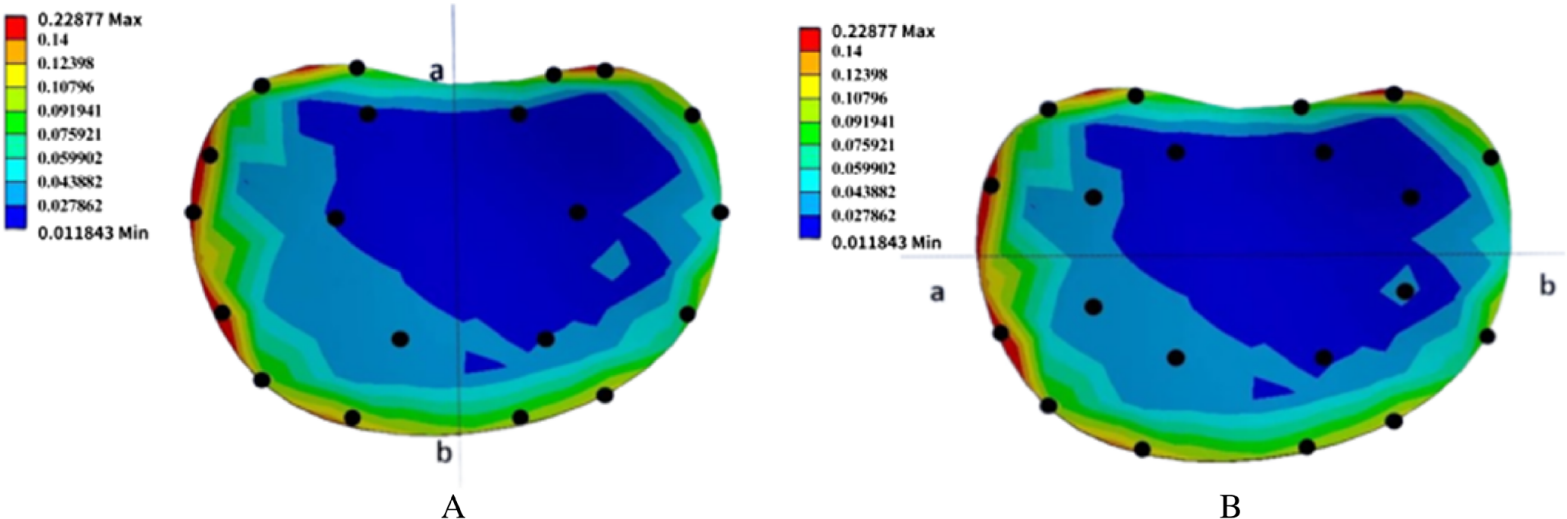
Schematic representation of the convex and concave lateral pressure points of the intervertebral disc. (A) Convex side and the concave side points of T1–T2 disc. (B) Anterior and posterior points of T1–T2 disc.

Second, the height ratios of the concave and convex sides of the upper and lower intervertebral spaces at the apex of vertebrae T7 and T2 (for T1–T2, T2–T3, T6–T7, and T7–T8) were measured and calculated. Third, the von Mises stress ratios for the concavities and convexities of the T1–T2, T2–T3, T6–T7, and T7–T8 discs were determined. The average value for the IVD stress was obtained by taking 10 points in the convex and concave, respectively (Figure 7) [56]. A larger concave/convex ratio of the height of the intervertebral space means that the concave side of the intervertebral space increased to create favorable conditions for disc remodeling. A smaller stress ratio for the concave side of the disc indicates that the originally overstressed concave side of the disc is released, breaking the vicious cycle of scoliosis progression by creating favorable remodeling through the disc. The ratio of axial forces in the concave and convex side muscles was measured. Based on the premise that the spinal skeleton returns to the midline, balanced axial force on the concave and convex sides of the muscle is beneficial to the construction of reasonable tensegrity.

## 3 Results

### 3.1 Radiological Parameter

The radiological parameters when different combinations of corrective forces were applied in the model are given in Table 5. In the coronal plane, the Cobb angle correction rates were highest in the MT curve and lumbar curve when the CPLL was 0.035 MPa (100% threshold), followed by 75%, 50%, and 25% thresholds. In contrast, the correction rates of the PT curve were 25%, 50%, 75%, and 100% in the order of high to low. The distance reduction sequences for AVT_T7_ were 100%, 75%, 50%, and 25%. The distance reduction sequences for AVT_T2_ were 25%, 50%, 75%, and 100%. The TK angle for the sagittal plane was 7.4° when the CPLL was 0.09 MPa, which was the maximum value at 25%. A TK angle of < 10° is diagnosed as a flatback deformity. The remaining correction force pressure combination of TK was in the range of 10°–40°. In the simulation, LL decreased when the correction threshold of the left lumbar region reached 50% and 75%, which was still in the normal physiological range.

**TABLE 5:** Simplified term.

Abbreviations	Full term
AIS	Adolescent idiopathic scoliosis
AVT	Apical vertebral translation
CAD	Computer‐aided design
CPLL	Correction pressure of the left lumbar region
CSVL	Center sacral vertical line
EO	Externus oblique
EOS imaging	
FEM	Finite element model
ICPL	Iliocostalis lumborum pars lumborum
ICPT	Iliocostalis lumborum pars thoracic
IO	Internus oblique
IP	Iliopsoas
IVD	Intervertebral disc
LGPL	Longissimus thoracis pars lumborum
LGPT	Longissimus thoracis pars thoracic
LL	Lumbar lordosis
MF	Multifidus
MT	Main thoracic
PT	Proximal thoracic
PM	Psoas muscle
QL	Quadratus lumborum
RA	Rectus abdominis
ROM	Range of motion
TK	Thoracic kyphosis

### 3.2 Intervals Height Concave to Convex Ratios

**FIGURE 8:**
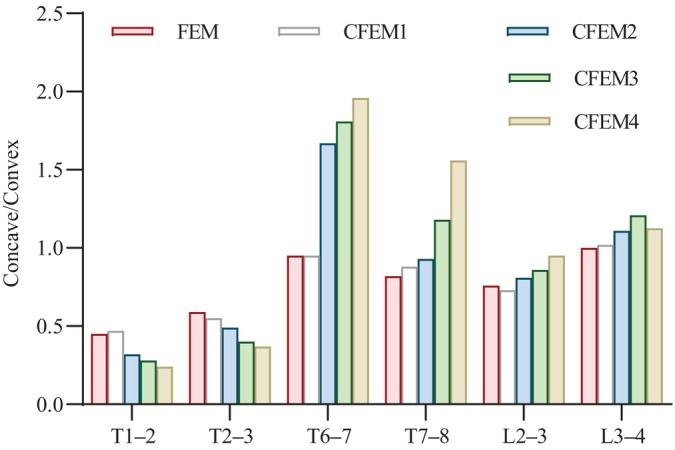
Concave/convex height ratios of intervertebral gaps under four simulated conditions.

Variations in the concave/convex height ratios in the upper and lower IVDs at the apical vertebra when simulating four combinations of corrective pressures are shown in Figure 8. For the concave–convex ratio of the intervertebral space height of the vertebrae at the apex of the PT curvature, the most satisfactory performance was achieved at 25% CPLL, followed by 50%, 70%, and 100%. In the MT region, the best performance was achieved when CPLL was 100%, followed by 75%, 50%, and 25%. Lumbar curve measurements were most satisfactory when the CPLL was 75%, followed by 100%, 50%, and 25%.

### 3.3 The von Mises Stress Ratios From Concave to Convex in IVDs

**FIGURE 9:**
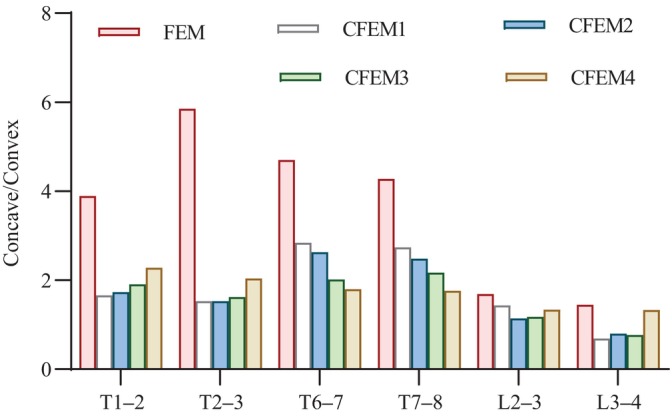
Concave/convex stress ratios of intervertebral discs under four simulated conditions.

The concave/convex stress ratios of the disc at the apex are shown in Figure 9. The most satisfactory performance in the concave/convex ratio of the upper and lower discs at the apex of the PT region was at 25% CPLL, followed by 75%, 50%, and 100% CPLL. At the apex of the MT, the most satisfactory performance was at 100% CPLL, followed by 75%, 50%, and 25% CPLL. The most satisfactory performance of the concave/convex ratio of the upper and lower discs at the apex of the lumbar curve was at 75% CPLL, followed by 50%, 100%, and 25%.

### 3.4 Muscles Axial Force in Concave to Convex Ratios

**FIGURE 10:**
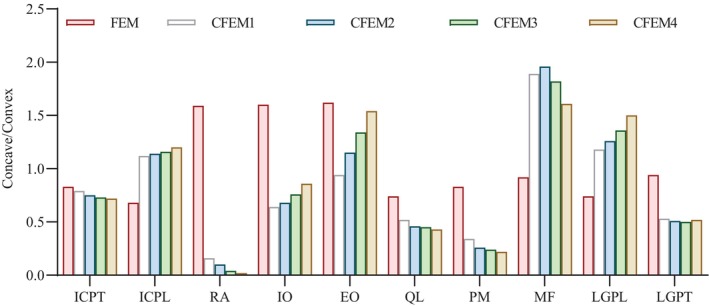
The concave/convex ratio of muscle axial stress under four simulation conditions. EO, external oblique; ICPL, iliocostalis lumborum pars lumborum; ICPT, iliocostalis lumborum pars thoracic; IO, internal oblique; LGPL, longissimus thoracis pars lumborum; LGPT, longissimus thoracis pars thoracic; MF, multifidus; PM, psoas muscle; QL, quadratus lumborum; RA, rectus abdominus.

Changes in the axial stress ratio of muscles on the concave and convex sides after different corrective pressures loading are shown in Figure 10. After the correction group was applied, the ICPL/IO/EO/LGPL tended to be balanced; when the CPLL boundaries were 25%, 50%, 75%, and 100%, the balance changed from best to worst. The other muscles were more unbalanced than before the correction in each correction combination, especially when the LGPT reached 100%. Conversely, imbalances occurred in the axial force ratio of muscles, including an increase in MF and a decrease in RA, QL, and PM.

## 4 Discussion

### 4.1 Main Findings

In this study, the FEM of “tensegrity” of AIS was constructed and effectively verified as the first innovative result of this study. Another unprecedented result from the simulation of brace intervention was that different combinations of corrective forces showed different characteristics in terms of radiological measurements, disc stress distribution and height, and muscle axial tension. Specifically, the combination with the highest correction pressure resulted in a high Cobb correction rate for the MT curve, whereas the correction rate for PT and the disc stress at the apex of PT and MT exhibited unsatisfactory characteristics. Interestingly, the biomechanical performance of Rigo A classification can be achieved by properly downregulating the correction force of the lumbar lateral curve. It was a combination of radiometric measurements, disc, and muscle axial force parameters that gave us evidence to support our initial clinical observations and hypotheses that were consistent. These results suggest that when brace intervention is used to break the vicious cycle, it should not only consider the correction rate of Cobb Angle, but also focus on the asymmetry of IVD and muscle axial force.

### 4.2 Verification for Tensegrity of FEM

The geometry of the EFM in this study is consistent with the radiological measurements, and the ROM verification of the main curve is close to the previous model, supporting the validity of the model. The ROM of the T1–T4 spinal segments was within a reasonable range and close to the ROM reported by previous authors [48, 49, 50]. The simulated T5–T8 spinal segment ROM in the left and right lateral flexion directions exhibited a 6° difference compared with the autopsy results of Busscher et al. [48] and the differences in the rest of the directions were in the range of 1°–2°. The experimental results were also similar but slightly lower than the results of Xin et al. [50] and Sheng et al. [49]. The differences may be related to the individual morphologic differences in age, gender, and spinal and other skeletal morphologic characteristics that affect joint mobility in AIS patients.

### 4.3 Simulation of Chêneau Brace Intervention

#### 4.3.1 Changes in Radiological Parameters During Simulation

Model simulations with brace corrections were performed to observe changes in radiological parameters. Like studies [49, 57], the main curve correction rate increased with increased correction pressure. However, this study adopted the Chêneau brace correction concept and set a specific correction area. The left armpit was elevated to produce antigravity and lateral flexion effects, which may also cause compensatory PT increases when the maximum correction pressure is reached.

According to the Rigo classification, Rigo A consists of three subtypes. Compared with A1 and A2, A3 has more functional waist bending. The waist bending arc is small but has a greater impact on the overall curve, so the design of the waist correction force for Rigo A3 bracing is particularly important [58]. The simulated corrected pressure reached the tolerance threshold of patients in the thoracic vertebra region, and only the corrected pressure in the left lumbar region changed in different correction combinations. The correction rate for the MT curve Cobb angle was low in this experimental simulation, except for a 54% correction rate when CPLL reached the maximum threshold. Simultaneously, a compensatory increase in PT curvature was observed. In general, the present model simulations exhibit unsatisfactory Cobb correction rates, similar to previous studies. For the lumbar curve, the correction was the greatest when the corrective force at the lumbar region reached 75% (CFEM3), which was rarely reported in previous studies. The maximum corrective stress of the secondary curve is not the primary consideration of this study and should be analyzed in conjunction with the corrective performance of the primary curve in future studies [18].

Overall, the correction of the Cobb angle was low in this simulation. However, the Cobb angle may be progressively increased by creep when the brace is worn. According to Périé et al. [36], the correction force in the finite element simulation only corrects the Cobb angle of the coronal plane by 9°, while the actual correction is 16°. Therefore, in addition to the passive correction force, the orthopedic mechanism of the brace may involve other factors that jointly promote mechanical balance in the brace. The simulation results of Andriacchi et al. [59] demonstrate that the brace cannot achieve a large immediate correction effect due to the relatively small corrective force applied in actual clinical practice. Andriacchi et al. also suggested that the corrective effect of the brace can be extended by spinal growth, gradual soft tissue relaxation, and sustained muscle activity after a few months of applied corrective force. Creep, which is an important concept in the treatment of scoliosis, refers to the deformation of a material under constant load that increases with time. When a constant corrective force is applied during the correction of spinal deformities, subsequent correction will occur due to creep [60]. Our model simplifies the biomechanical changes in vivo while wearing the brace but does not simulate the creep of the musculoskeletal system or the changes in tension or support force caused by creep of the brace and brace materials. These differences may affect the correction rates of the brace.

In our model, the displacement (AVT) of T2 and T7 on the *X*‐axis is consistent with the correction force direction of the main chest curve, resulting in T2 being further away from the CSVL line and T7 being closer to the CSVL line than the original model. Thus, wearing braces can improve the Cobb angle of the main chest curvature in AIS patients, but may affect the standing balance or lead to effects similar to the Crankshaft phenomenon in AIS patients after surgery. With the downward adjustment of CPLL pressure, the correction rate of the MT curve is decreased, but the correction rate of PT curve does not decrease. The biological characteristics of the IVDs with the double thoracic curve are more reasonable, which is more conducive to reversing the vicious cycle law of Stoke and Hutter. Reversing this cycle may increase spinal growth in the central axis in teenagers. These results suggest that a better coronal balance can be obtained by adjusting the corrective force of the left lumbar lateral curvature area appropriately.

The correction simulation did not cause significant changes in TK. The most significant change was an increase of only 3.3°–14.3° when the CPLL pressure was at 50% of the maximum threshold. These results suggest that the sagittal curve was not reshaped in the simulation. When the CPLL pressure was 100%, the Cobb correction rate was the highest, but TK was not affected. Like previous studies, achieving optimal correction may cause a flat back deformity. The risk of a flat back may be reduced by adjusting the corrective force coupling [17]. Nonetheless, high‐quality evidence supporting improvements in flat back remodeling of the sagittal curve using the Chêneau brace is lacking, and flat back remodeling remains a challenge [58].

Matsumoto et al. [61] showed that AVT is closely related to the postoperative distal attachment phenomenon. Liu et al. [62] investigated the correlation between three‐dimensional spinal–pelvic parameters and standing balance and gait characteristics in AIS and showed that the standing balance of Lenke V AIS patients is influenced by their AVT, PT, and pelvic axial rotation.

#### 4.3.2 Changes in IVD Parameters During Simulation

The height of IVD, which is related to the height of intervertebral space, is the main reason for changes in the vertebral body height. Therefore, understanding the height of concavity and convexity in the intervertebral space and the distribution of IVD stress is crucial for the long‐term effectiveness of scoliosis correction. The clinical observations of de Reuver et al. [63] showed that the height of the concave side of the spine was smaller than the height of the convex side at the curve apex.

Differential growth of the spine may occur within the IVD first. The growth is manifested as early wedge‐shaped changes in the IVD, ending with sudden growth and severe vertebral deformation [6]. Therefore, the height and stress parameters of the disc should be considered when wearing the corrective stent. To our knowledge, few finite element analyses of these problems have been reported. Our study shows that progressively decreasing the corrective force in the lumbar region can ameliorate the concave–convex height asymmetry in the parietal space near the thoracic curve. When the correction force of the lumbar curve was gradually increased to the maximum, the concave/convex space ratio of the thoracic apex curve gradually increased. The same pattern was observed in the stress values of the convex side of the IVD. Thus, when the maximum correction force of the lumbar curve reaches the upper limit of the threshold, the correction rate of the chest curve is optimal and may break the vicious cycle. However, the compensatory increase in the PT curve should be considered. Gradually reducing the correction force of the lumbar curve may prevent the compensatory aggravation of the near thoracic curve, but the correction rate of the MT curve will decrease. Improving the concavity‐to‐convexity ratio in intervertebral space height means that the asymmetric stress on both sides of the concavity and convexity during IVD growth in AIS tends to be balanced. According to Hutte Volkman's Law, this may break the vicious cycle of asymmetric growth of the spine and improve the deformity.

#### 4.3.3 The Change of Muscle Axial Force During Simulation

We simulated the forces of the muscles that stabilize the spine. Our model was similar to the model of Kamal and Rouhi [64] in that the axial force on the convex side of the muscle was greater than the force on the concave side. This may be due to a compensatory change in the muscle to maintain the mechanical stability of the spine. The more the curve deviates from the center line, the more unbalanced the trunk muscle force [65, 66].

In general, ICPL/IO/EO/LGPL tend to be balanced in simulation corrections. When the CPLL pressure gradually decreases, this trend is clearer. Synchronous, LGPT, MF, RA, QL, and PM are still out of balance, which may further explain the complexity of scoliosis deformity. The cumulative force of the multifidus muscle is increased by approximately 27%. Our model simulated a more pronounced abdominal muscle imbalance during brace correction. This can be explained by Stoke's theory. To maintain the mechanical stability of the spine, antagonistic muscles in the abdomen are activated to strengthen the spine during continuous load tasks with different heights, constant posture, and external torque [67].

The balance constraint allocates more force to the active muscles in the back to maintain a balanced state. When corrective force is applied to open the concave and convex sides of the spine, the muscles on the concave side of the back are activated to maintain balance and the axial force of the muscles increases. Other electromyography and optimized models indicate that the spine is more stable in more demanding activities. However, the finite element simulation conducted by Hajihosseinali et al. [18] demonstrated that coactivation occurred in the abdominal muscles, unlike other in vivo studies. This is consistent with our experimental results. The correction force of the lumbar curve is reduced to obtain a more balanced representation of the overall radiology, disc, and muscle axial force. We believe this simulation can be used to design more effective conservative clinical treatments for patients with AIS. Specifically, the pros and cons of disc and radiographic parameters should be weighed when facing a double thoracic curve. If asymmetry of muscle axial force occurs under a relatively reasonable central axis after wearing the brace, physical therapy intervention may be needed.

### 4.4 Limitations and Strengths

To our knowledge, this study is the first to developmentally construct a musculoskeletal FEM that embodies the tensegrity of the spine as a brace intervention simulation for AIS. It is possible to obtain excellent biomechanical performance by reducing the correcting force of the compensation curve. Musculoskeletal models fitted with biomechanical characteristics may enrich the multidimensional thinking of clinical intervention with braces. There are several limitations to this study. First, the effects of immediate correction of the brace were modeled, and the effects of long‐term correction of the brace were not analyzed. Immediate correction may correlate with long‐term treatment. However, to obtain a more detailed long‐term treatment effect, future studies should include dynamic simulations of individual growth. Second, no complete soft tissue model such as muscle was available to compare and validate the model. This experiment did not construct tissues such as internal organs and did not fully model the effects of human internal organs on the axial forces of spinal bones, IVDs, and muscles. Finally, the effects of individual patient differences cannot be excluded. In the future, a larger number of patients with more classifications will be included to enhance the generality of the findings.

### 4.5 Prospect of Clinical Application

Our simulation study has implications for brace correction in patients with challenging multiple curves. Theoretically, the design of the brace would be different depending on the type of scoliosis. As more scoliosis models are developed, the biomechanical effects of different types of scoliosis braces should be analyzed to improve the predictability of clinical outcomes.

## 5 Conclusion

The FEM of AIS patients including musculoskeletal is effectively established, which provides a numerical platform for the correction of the Chêneau brace. Downregulating CPLL pressure may improve the radiological parameters and biomechanical manifestations of the thoracic curve. Muscle imbalances that occur during bracing may be a concern for physical therapy.

## Author Contributions

**Xiaohui Zhang:** writing review and editing, writing original draft, validation, software, methodology, formal analysis, data curation, and conceptualization. **Di Wang:** writing review and editing. **Danyu Lv:** writing review and editing, resources. **Jinmiao Lv:** writing review and editing, writing original draft, validation, formal analysis, conceptualization. **Bagen Liao:** writing review and editing, writing original draft, validation, supervision, formal analysis, conceptualization. **Huiyi Tang:** writing review and editing. **Jinlin Qian:** writing original draft.

## Ethics Statement

The study was conducted according to the guidelines of the Declaration of Helsinki and approved by the Scientific Board of Guangzhou Sport University (2021LC LL‐16).

## Consent

Written informed consent to participate in this study was provided by the participantsandapos.legal guardian written informed consent was obtained from the individual.

## Conflicts of Interest

The authors declare no conflicts of interest.

## Data Availability

Data for this study are available upon request as the data contained patient privacy and other sensitive information. Data requests can be sent to corresponding author (bagenliao@sina.com) and obtained after obtaining informed consent from the patient.
